# High-risk landscapes of Japanese encephalitis virus outbreaks in India converge on wetlands, rain-fed agriculture, wild Ardeidae, and domestic pigs and chickens

**DOI:** 10.1093/ije/dyac050

**Published:** 2022-03-31

**Authors:** Michael G Walsh, Amrita Pattanaik, Navya Vyas, Deepak Saxena, Cameron Webb, Shailendra Sawleshwarkar, Chiranjay Mukhopadhyay

**Affiliations:** Faculty of Medicine and Health, School of Public Health, The University of Sydney, Camperdown, New South Wales, Australia; Faculty of Medicine and Health, Sydney Institute for Infectious Diseases, The University of Sydney, Westmead, New South Wales, Australia; One Health Centre, The Prasanna School of Public Health, Manipal Academy of Higher Education, Manipal, Karnataka, India; The Prasanna School of Public Health, Manipal Academy of Higher Education, Manipal, Karnataka, India; Manipal Institute of Virology, Manipal Academy of Higher Education, Manipal, Karnataka, India; One Health Centre, The Prasanna School of Public Health, Manipal Academy of Higher Education, Manipal, Karnataka, India; The Prasanna School of Public Health, Manipal Academy of Higher Education, Manipal, Karnataka, India; Department of Epidemiology, Indian Institute of Public Health Gandhinagar, Gandhinagar, Gujarat, India; Faculty of Medicine and Health, Sydney Institute for Infectious Diseases, The University of Sydney, Westmead, New South Wales, Australia; Department of Medical Entomology, NSW Health Pathology, Westmead Hospital, Westmead, New South Wales, Australia; Faculty of Medicine and Health, Sydney Institute for Infectious Diseases, The University of Sydney, Westmead, New South Wales, Australia; One Health Centre, The Prasanna School of Public Health, Manipal Academy of Higher Education, Manipal, Karnataka, India; The Prasanna School of Public Health, Manipal Academy of Higher Education, Manipal, Karnataka, India; The University of Sydney, Faculty of Medicine and Health, Westmead Clinical School, Westmead, New South Wales, Australia; Department of Microbiology, Kasturba Medical College, Manipal Academy of Higher Education, Manipal, Karnataka, India; Centre for Emerging and Tropical Diseases, Kasturba Medical College, Manipal Academy of Higher Education, Manipal, Karnataka, India

**Keywords:** Zoonosis, vector-borne disease, landscape epidemiology, wildlife–livestock–human interface, Japanese encephalitis

## Abstract

**Background:**

Japanese encephalitis virus (JEV) is a zoonotic mosquito-borne virus that causes a significant burden of disease across Asia, particularly in India, with high mortality in children. JEV circulates in wild ardeid birds and domestic pig reservoirs, both of which generate sufficiently high viraemias to infect vector mosquitoes, which can then subsequently infect humans. The landscapes of these hosts, particularly in the context of anthropogenic ecotones and resulting wildlife–livestock interfaces, are poorly understood and thus significant knowledge gaps in the epidemiology of JEV persist. This study sought to investigate the landscape epidemiology of JEV outbreaks in India over the period 2010–2020 to determine the influence of shared wetland and rain-fed agricultural landscapes and animal hosts on outbreak risk.

**Methods:**

Using surveillance data from India’s National Centre for Disease Control Integrated Disease Surveillance Programme, JEV outbreaks were modelled as an inhomogeneous Poisson point process and externally validated against independently sourced data.

**Results:**

Outbreak risk was strongly associated with the habitat suitability of ardeid birds, both pig and chicken density, and the shared landscapes between fragmented rain-fed agriculture and both river and freshwater marsh wetlands.

**Conclusion:**

The results from this work provide the most complete understanding of the landscape epidemiology of JEV in India to date and suggest important One Health priorities for control and prevention across fragmented terrain comprising a wildlife–livestock interface that favours spillover to humans.

Key Messages

- This is the first investigation of Japanese encephalitis virus (JEV) outbreak risk in India to show a critical convergence of natural wetland habitat with rain-fed agriculture and wildlife and domesticated animal reservoirs.
- Associations between JEV outbreaks and the biotic and abiotic environment, and particularly the effect modification of rain-fed agriculture by wetland habitat and vice versa, demonstrated the potential influence of anthropogenic ecotones in demarcating risk.
- By focusing on landscape, this investigation provides the most complete understanding of JEV epidemiology in India to date and identifies unique, multi-tiered transdisciplinary targets for prevention and control.

## Introduction

Japanese encephalitis virus (JEV) is one of the most substantial causes of childhood encephalitis in Asia.^1^ Although most infections are asymptomatic or mild (∼1 in 250 infections present with severe clinical disease), mortality is high among those presenting with encephalitis.^1^ In India, a country with a high burden of disease caused by JEV, 13.7% of 63 854 acute encephalitis cases from 2010 to 2017 were due to JEV and >17% of these cases died.^2^ Although the annual occurrence of Japanese encephalitis (JE) is high, there is considerable heterogeneity in its occurrence across the country with the north-east being a perennial hotspot for outbreaks, although additional far-removed areas of intractable endemicity also persist.^2^ Japanese encephalitis virus is a mosquito-borne zoonotic *Flavivirus* with enzootic and endemic transmission in animal and human hosts, respectively, although such baseline transmission is regularly punctuated with more substantial outbreaks.^3^^,^^4^ Outbreaks in India are generally seasonal following monsoon flooding, but transmission can and does happen at any time of the year with rural populations typically at highest risk, although some urban locations also experience outbreaks.^2^

The infection ecology of JEV is complex and incompletely understood in many landscapes within India. As a result, viral transmission is often poorly controlled. *Culex tritaeniorhynchus* is the most important vector for JEV across Asia^4^^,^^5^ and has a wide distribution in India.^5^^,^^6^ In addition to this highly efficient vector, there are at least four other important vectors (*Cx. vishnui*, *Cx. gelidus*, *Cx. fuscocephala* and *Cx. pseudovishnui*) that also exhibit wide distribution across South and Northeast India.^6^^,^^7^ Given the wide range of suitable habitats for these mosquitoes, exposure to JEV vectors is extensive throughout the country. Wading bird species in the Ardeidae family are the primary reservoirs and maintenance hosts for JEV,^8–11^ whereas domestic pigs are key amplifying hosts that frequently accelerate spillover to humans.^12–17^ This general distinction between host groups notwithstanding, high viraemias have been shown in several Ardeidae species, so these maintenance hosts may also simultaneously act as amplifying or bridging hosts, depending on the nature of their interface with humans or pigs.^4^ Moreover, some heron species can readily adapt to some agricultural practices (e.g. rice paddies), increasing contact with people and domestic animals in these settings.^18^ Interestingly, specific maintenance host–mosquito vector–amplifying host interactions have been identified showing *Cx. tritaeniorhynchus* zoophilic feeding preferences for herons and domestic pigs, which may further highlight the importance of interface and the potential for an efficient bridge to human spillover in landscapes that favour these interactions.^4^ Although pigs have been demonstrated as key amplifying hosts for JEV, some evidence suggests that poultry may also act as bridging hosts to human spillover in some settings.^11^^,^^19^^,^^20^ However, poultry are frequently ignored in relation to human outbreaks and so their role in JEV epidemiology represents an additional knowledge gap. Human infections, although they can present with severe disease, typically do not generate sufficiently high viraemia to infect the mosquito vectors and so humans, as dead-end hosts, do not contribute meaningfully to virus circulation.^4^

**Figure 1:**
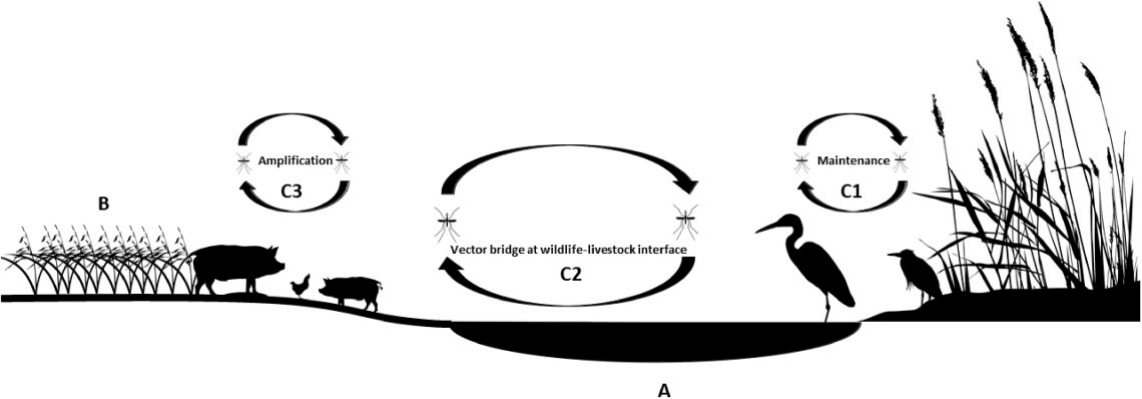
Theoretical representation of landscapes with wetland (a) and rain-fed crops (b) and their potential animal host occupants. Multiple transmission cycles of Japanese encephalitis virus (JEV) may be realised in such landscapes such as transmission among Ardeidae maintenance hosts (C1), shared transmission between ardeid birds and domestic pigs and chickens at the wildlife–livestock interface (C2) and concentrated transmission among domesticated amplification hosts (C3).

The biotic factors described above define the vectors and hosts in which JEV circulates and the nature of interspecies interaction that may drive viral transmission dynamics in hosts, but there are equally important abiotic factors that can influence JEV transmission such as wetlands and rain-fed agriculture. Heterogeneous wetlands not only provide a spectrum of favourable habitat for vectors, they also demarcate critical habitat for key ardeid reservoirs.^21^ Rain-fed agriculture tends to comprise agricultural systems that (i) are engaged by poorer, subsistence communities and (ii) exhibit limited or no control of water distribution in the landscape.^22^ Both wetland habitat and rain-fed agriculture can influence mosquito habitats by shaping the distribution of water in the landscape and since both landscapes can be sensitive to the modulating effects of climate, these could represent important vector foci.^23^ Moreover, rain-fed crops that lie within or adjacent to wetland habitat may present ecotones (i.e. areas of spatial transition between ecological communities or ecosystems) of particular risk since these often also present landscapes occupied by key animal hosts and may therefore exhibit multiplicities of JEV transmission (Figure 1). In India, although some states have been recognized as hotspots of annual JEV outbreaks, the landscape epidemiology of JEV has not been thoroughly described in these and other areas of occurrence. The heterogeneity of risk is particularly noteworthy since viable mosquito vectors can be found in most parts of the country. As such, the delineation of JEV outbreak risk across India requires a broader consideration of diverse landscapes, representing shared configurations of wetland habitat, rain-fed agriculture and animal hosts (Figure 1).

**Figure 2:**
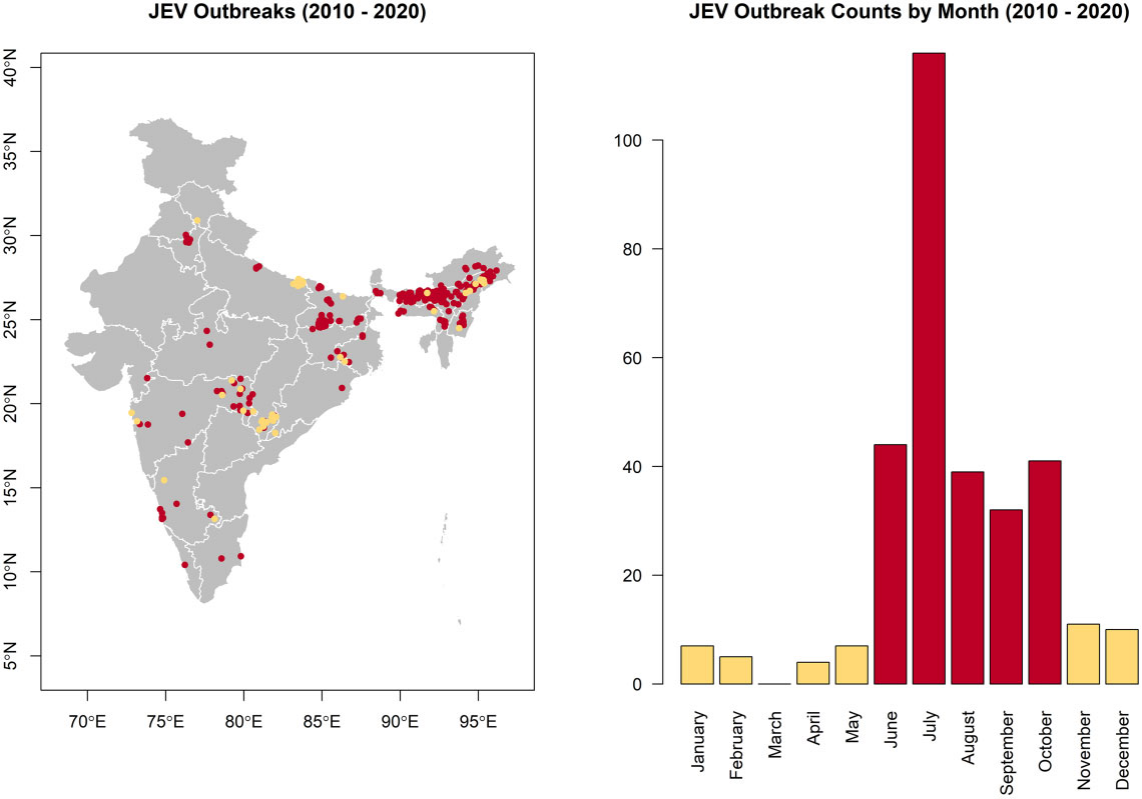
The spatial (left) and temporal (right) distributions of Japanese encephalitis virus (JEV) outbreaks in India. Outbreaks that occurred during the high incidence period are represented in dark shade (burgundy) and those that occurred during the low incidence period in light shade (khaki). The map does not reflect the authors’ assertion of territory or borders of any sovereign country including India and is displayed only to present the distribution of JEV occurrence.

The current study sought to identify the key landscape features of JEV outbreaks in India. In particular, this investigation examined the associations between JEV occurrence in humans and the distribution of maintenance and amplifying animal hosts, wetland hydrogeography and flow dynamics, rain-fed agriculture and climate. Although considerable heterogeneity in risk was anticipated, it was hypothesised that river wetlands and rain-fed agriculture with high pig density and high Ardeidae suitability would drive the landscape epidemiology of JEV.

## Methods

A brief summary of the methods is provided below, whereas a complete detailed description of all data sources and modelling procedures is provided in the Supplementary Methods (available as Supplementary data at *IJE* online).

### Data sources

#### Human data

The National Centre for Disease Control's Integrated Disease Surveillance Programme (IDSP) maintains ongoing surveillance of JEV infections under the administration of India's Ministry of Health and Family Welfare.^24^ There were 294 laboratory-confirmed and location-unique outbreaks of JEV reported at the village level (spatial resolution of 1 arc minute, or ∼2 km) to the IDSP between 1 January 2010 and 31 December 2020 (Figure 2). These were included as the primary training data in the current study. A secondary data set (*N* = 27) comprising all available independent, laboratory-confirmed community surveys of human and mosquito infection conducted within the same time period as the IDSP surveillance and with published location data was used to test the external validity of these surveillance data.^25–28^

This study adjusted for potential reporting bias of JEV infections using the distribution of health system performance as a representation of the local capacity to detect cases. The infant mortality ratio (IMR) was chosen as a proxy for health system performance since it has been validated as representative of health infrastructure and health system performance, and used to assess health service delivery and performance in diverse settings.^29–31^ Human population density was derived from the Gridded Population of the Word estimates for the 2010 population to represent the baseline population at the beginning of the period under study.^32^

#### Animal data

The Global Biodiversity Information Facility (GBIF) was used to acquire all observations of Ardeidae species (241 784 individual observations of 15 species; Supplementary Tables and Figures: Supplementary Table S1, available as Supplementary data at *IJE* online) between 1 January 2010 and 31 December 2020 across India so each species' distribution could be modelled.^33^ Due to potential differential accessibility, the background points used to model Ardeidae species distributions were weighted by the human footprint (HFP) (see modelling description below) to correct for potential spatial reporting bias in the observations of these birds.^34^ Pig, chicken and duck density data were obtained from the Gridded Livestock of the World^35^ (GLW).

#### Environmental data

Water movement through the landscape was quantified using hydrological flow accumulation obtained from the Hydrological Data and Maps based on SHuttle Elevation Derivatives at multiple Scales (HydroSHEDS) information system.^36^ Wetlands were classified using the surface water data from the Global Lakes and Wetlands Database.^37^^,^^38^ Agriculture data were obtained from the Global Food Security Support Analysis Data project to describe the geographic extent of crops that employ rain-fed water distribution systems.^39^ Two primary classes of rain-fed agricultural systems were represented: dominant rain-fed crops and fragmented rain-fed crops. Climate data were obtained from the WorldClim Global Climate database.^40^ All raster data described above were obtained at a resolution of 30 arc seconds.

#### Statistical analyses

##### Ardeidae species distribution modelling

An ensemble approach comprising boosted regression trees (BRT), random forests (RF) and generalised additive models (GAM) was used to estimate the landscape suitability of each of the 15 Ardeidae species. Model performance, based on the area under the receiver operating characteristic curve (AUC), and model fit, based on the deviance, were used to evaluate each of the three species distribution model (SDM) frameworks (BRT, RF and GAM) for each ardeid species. Subsequently, an ensemble landscape suitability was estimated for each species from the three SDM frameworks using their weighted mean, with weights based on the AUC.^41^ Potential spatial sampling bias in the GBIF database was adjusted for by sampling background points proportional to the human footprint as a proxy for landscape accessibility. After modelling the distributions of individual Ardeidae species’ landscape suitability, a composite of ardeid suitability was calculated based on the mean of all individual species suitability distributions.

##### JEV outbreak modelling

The JEV outbreaks were fit as a point process using homogeneous and inhomogeneous Poisson models.^42^ The models’ background points were sampled proportional to IMR, as described above, to control for potential reporting bias in the JEV infection surveillance. The crude associations between JEV outbreaks and each landscape feature were initially assessed individually using a separate simple inhomogeneous Poisson model (Supplementary Table S2, available as Supplementary data at *IJE* online). Features demonstrating bivariate associations with confidence intervals that did not include 0 were included as covariates in the multiple inhomogeneous Poisson models (Supplementary Figures S1–S3, available as Supplementary data at *IJE* online)). Interaction between fragmented rain-fed agriculture and the two dominant wetland types, freshwater marsh and river, were examined separately using a freshwater marsh–rain-fed crops model and a river–rain-fed crops model with a corresponding interaction term included in each model, respectively. In this way, the interaction between fragmented rain-fed agriculture and both freshwater marsh and river wetlands was used to evaluate the impact of their shared landscapes on JEV risk. The Akaike information criterion (AIC) assessed model fit, whereas the AUC assessed model performance. Importantly, model performance was tested against an independent, laboratory-confirmed data set derived from the community-based surveys described above, thus providing a test of the external validity of the results. Model selection was based on a comparison of the fit (based on AIC) of the full model to reduced model groups nested on three broad environmental domains (hydrogeography, animal hosts and climate). Assessment of K-functions fitted to the JEV outbreaks before and after point process modelling with the specified environmental features was used to determine whether the selected features adequately accounted for the observed spatial dependencies.

The silhouette images of ardeid birds, pigs, chickens, mosquitoes and rice in Figure 1 were acquired from phylopic.org and used under the Creative Commons licence.

## Results

The landscape suitability of individual Ardeidae species demonstrated a high degree of overlap with the composite landscape suitability (niche overlap >88% for all species and >96% for all but one species; Supplementary Table S1, available as Supplementary data at *IJE* online)), so the composite measure of Ardeidae suitability was used in the modelling of JEV outbreaks.

**Figure 3:**
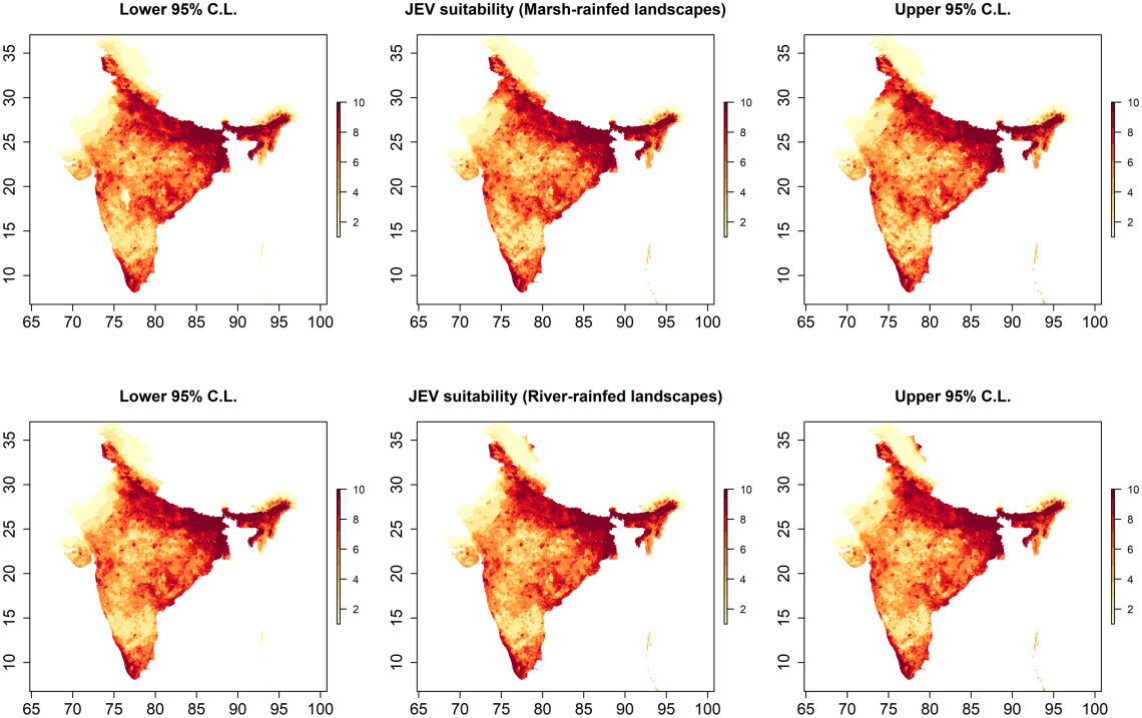
Japanese encephalitis virus (JEV) outbreak risk based on predicted intensity at 1.0 arc minutes (∼2 km). The centre panels depict the distribution of JEV risk for freshwater marsh-fragmented rain-fed agriculture (top) and for river-fragmented rain-fed agriculture (bottom) models as deciles of the predicted intensities from the best fitting and performing inhomogeneous Poisson point process models (Table 1). The left and right panels depict the lower and upper 95% confidence limits, respectively, for the predicted intensities. The map does not reflect the authors’ assertion of territory or borders of any sovereign country including India and is displayed only to present the distribution of JEV occurrence.

**Figure 4:**
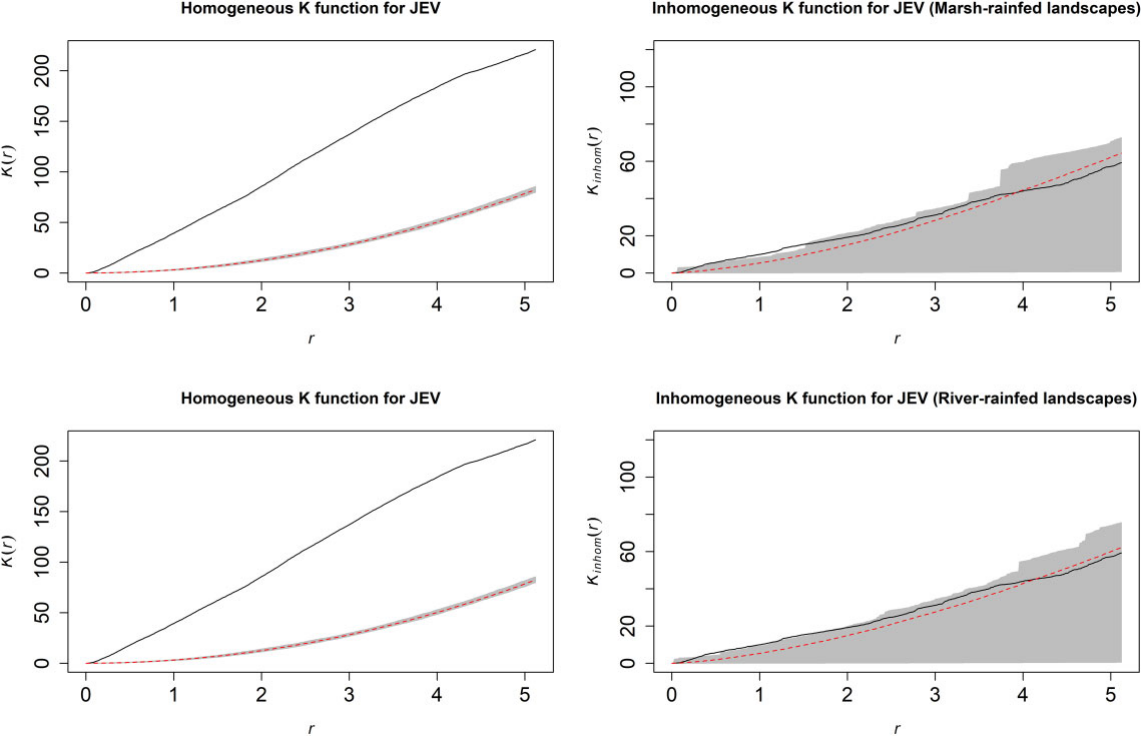
Homogeneous (left panels) and inhomogeneous (right panels) K-functions for the Japanese encephalitis virus (JEV) outbreak point process. The homogeneous K-function is not an appropriate fit due to the spatial dependency in JEV outbreaks as depicted by the divergent empirical (solid line) and theoretical functions (the latter is the theoretical function under complete spatial randomness, represented by the dashed line with confidence bands in grey). In contrast, the freshwater marsh-fragmented rain-fed agriculture (top) and river-fragmented rain-fed agriculture (bottom) model-based inhomogeneous K-functions show that the spatial dependency was accounted for by the model covariates (overlapping empirical and theoretical functions). The *x*-axes, *r*, represent increasing radii of subregions of the window of JEV outbreaks, whereas the *y*-axes represent the K-functions.

The best fitting and performing models of JEV outbreak risk included all variables except temperature and duck density under both landscape Scenario 1 (fragmented rain-fed crops with freshwater marsh wetlands) and landscape Scenario 2 (fragmented rain-fed crops with river wetlands). These final models presented in Table 1 correspond to reduced Models 7 and 8 in Supplementary Table S3, available as Supplementary data at *IJE* online). The final models were further corroborated by the stepwise selection procedure implemented using the full point process models. JEV outbreaks were strongly associated with Ardeidae suitability (Table 1, Model 1—relative risk (RR)  = 2.77, 95% CI 1.15–6.69; Model 2—RR = 2.44, 95% CI 1.02–5.86), pig density (Model 1—RR = 1.30, 95% CI 1.22–1.39; Model 2—RR = 1.29, 95% CI 1.20–1.38) and chicken density (Model 1—RR = 1.09, 95% CI 1.03–1.15; Model 2—RR = 1.09, 95% CI 1.03–1.16) whereby an increasing presence of each in the landscape was associated with increased risk. Proximity to fragmented rain-fed agriculture (Table 1, Model 1—RR = 0.976, 95% CI 0.968–0.985 and Model 2—RR = 0.978, 95% CI 0.970–0.987) was associated with increased risk of JEV outbreaks (inverse associations indicate that increasing distance from this feature was associated with decreasing risk and vice versa) but not proximity to major non-fragmented rain-fed agricultural systems (Supplementary Table S2, available as Supplementary data at *IJE* online). Importantly, proximity to both river and freshwater marsh wetlands was also strongly associated with increased risk and each modified the association between JEV outbreaks and fragmented rain-fed crops such that proximity to rain-fed agriculture was associated with greatest risk in locations where these crops were shared with, or adjacent to, the two wetland habitats and risk decreased with increasing distance from wetlands even as proximity to rain-fed crops remained constant (Table 1). The reverse, of course, is also true: proximity to rain-fed agriculture also modified the associations between JEV outbreaks and proximity to the wetland habitats. As expected, climate, especially precipitation, was also strongly associated with JEV outbreaks. Estimates of the distribution of JEV outbreak risk with 95% confidence limits are presented in Figure 3. The spatial dependency apparent in JEV outbreaks as estimated by the homogeneous K-function (Figure 4, left panels) was largely accounted for by the final inhomogeneous Poisson models (Figure 4, right panels).

**Table 1:** Adjusted relative risks and 95% confidence intervals for the associations between Japanese encephalitis virus outbreaks and each landscape feature as derived from the best fitting inhomogeneous Poisson models. Each landscape feature is adjusted for all others in each of the two models.

Landscape feature	Relative risk	95% confidence interval	*P*-value
**Model 1: Freshwater marsh–rain-fed crops interaction**			
Ardeidae landscape suitability (%)	2.77	1.15–6.69	0.01
Pig density (deciles)	1.30	1.22–1.39	<0.00001
Chicken density (deciles)	1.09	1.03–1.15	0.003
Distance to river (km)	0.997	0.995–0.999	0.005
Distance to freshwater marsh (km)	0.996	0.995–0.997	<0.00001
Distance to fragmented rain-fed agriculture (km)	0.976	0.968–0.985	<0.00001
Freshwater marsh:fragmented rain-fed agriculture	1.00008	1.00005–1.0001	<0.00001
Mean precipitation during the wettest quarter (10 cm)	1.008	1.006–1.009	<0.00001
Mean precipitation during the driest quarter (10 cm)	1.15	1.10–1.20	<0.00001
**Model 2: River–rain-fed crops interaction**			
Ardeidae landscape suitability (%)	2.44	1.02–5.86	0.0002
Pig density (deciles)	1.29	1.20–1.38	<0.00001
Chicken density (deciles)	1.09	1.03–1.16	0.001
Distance to river (km)	0.996	0.994–0.998	0.00007
Distance to freshwater marsh (km)	0.997	0.996–0.998	<0.00001
Distance to fragmented rain-fed agriculture (km)	0.978	0.970–0.987	<0.00001
River:fragmented rain-fed agriculture	1.0001	1.00007–1.0002	<0.00001
Mean precipitation during the wettest quarter (10 cm)	1.007	1.006–1.009	<0.00001
Mean precipitation during the driest quarter (10 cm)	1.15	1.10–1.20	<0.00001

## Discussion

This is the first investigation of JEV outbreaks to consider the impact of shared landscapes with key wildlife and domesticated animal reservoirs for JEV while simultaneously assessing the convergence of natural wetland habitat with rain-fed agriculture. Wild ardeid and domestic pig and chicken hosts were strongly associated with JEV outbreak risk. River and freshwater marsh systems and their shared landscapes with fragmented rain-fed agriculture were also strongly associated with outbreak risk. This configuration of landscape features presents the most substantive evidence to date of ecotonally-driven risk for JEV outbreaks in India and could have potentially important policy implications across multiple disciplines and sectors for the control and prevention of outbreaks. For example, some features may be best mitigated by local municipalities due to their highly localised context, such as the sharing of space between ardeid birds and domestic animals, whereas features requiring more resource-intensive measures, such as veterinary surveillance of pigs and chickens, may be more effectively targeted and resourced by state or national authorities.

The family Ardeidae comprises the wading birds, herons (including egrets) and bitterns. Ardeid birds have been recognised as key maintenance hosts for JEV.^8–11^ Domestic pigs, conversely, are important amplification hosts due to the high viraemia associated with porcine infection.^12–17^ Pigs are also important since these livestock animals typically occupy space in close proximity to humans, although several heron species, such as the cattle egret, *Bubulcus ibis*, are also capable of thriving in anthropogenic landscapes.^43^ Therefore, as expected, both ardeid birds and domestic pigs were strongly associated with outbreak risk in the current study. Importantly, this study also highlighted an association between chickens and humans spillover, which is noteworthy since poultry are frequently overlooked in domesticated animal surveillance for JEV despite previous investigations identifying chickens as viable hosts.^11^^,^^19^^,^^20^^,^^44^ As such, JEV surveillance could benefit from the inclusion of chickens in monitoring programmes rather than focusing on pigs alone, the stronger influence of the latter notwithstanding. Importantly, the current study did not observe and assess specific interactions between ardeid birds and pigs or chickens across India, which precludes any definitive conclusions about the roles of these hosts in the infection ecology of JEV. Field investigations of interspecific interactions in local settings will be required to verify the results from the current study and ultimately define how different classes of hosts operate with respect to viral circulation and spillover.

Wetlands can provide important habitat for mosquitoes and therefore increased outbreak risk associated with the provision of a stable source of surface water in these habitats is intuitive. Nevertheless, wetland systems are not homogeneous geomorphologically or ecologically, and neither were they homogeneous with respect to JEV occurrence as clearly demonstrated by the lack of association between outbreak risk and proximity to any surface water type (Supplementary Table S2 (available as Supplementary data at *IJE* online)). Instead, river and freshwater marsh wetlands dominated JEV outbreak risk, with both also demonstrating interaction with fragmented rain-fed agriculture suggesting that shared landscapes of wetland habitat and fragmented rain-fed crops may be particularly important to the landscape epidemiology of JEV outbreaks. These associations are intuitive because mosaics of wetlands and rain-fed agriculture may represent landscapes of more seasonally stable precipitation or water availability compared with rain-fed crops that are far removed from wetland habitat. Furthermore, fragmented rain-fed agriculture within or adjacent to wetlands may also demarcate landscapes with limited control of water dispersal following inundation,^22^ which is particularly relevant to the annual monsoon flooding and which corresponds to the season of highest JEV incidence. It is also important to note that rain-fed agriculture is typically a system employed by resource-limited subsistence farmers, with fragmented agricultural landscapes often corresponding to more economically disadvantaged communities^22^ and which also tend to represent a preponderance of the annual JEV incident cases.^2^ Therefore, not only do these findings provide further insight into the epidemiology of JEV outbreaks, they also identify vulnerable communities that are likely to be at greatest risk and which may yield maximum benefit from targeted resource allocation to prevent future outbreaks.

As expected, increasing precipitation was associated with increased JEV outbreak risk. Interestingly, temperature was inversely associated with JEV outbreak risk bivariately, but when considered in the multiple point process models with the other landscape features, the association did not persist. Future work will need to explore the effects of specific weather events and patterns with the requisite temporal resolution to link fluctuations in precipitation and temperature with individual JEV outbreaks. For example, one study examined a long-term time series of JEV occurrence and found that increases in both rainfall and temperature were associated with increased risk.^23^ However, this work was limited to one district in one state, so more work will require examination across many more of India’s heterogeneous landscapes to better understand how weather fluctuation may operate in different landscapes. Nevertheless, the association between JEV and precipitation has shown broad geographical consistency as manifested in China, for example, where cases were mostly concentrated in landscapes with annual precipitation of >400 mm irrespective of whether these landscapes were characterised by warm-temperate, semi-tropical or tropical climate regimes.^45^

It is important to acknowledge and discuss some additional limitations attending this work. First, although the national IDSP surveillance system was used to capture all reported outbreaks under investigation, we recognise that reporting bias may still be present. To correct for potential reporting bias, rather than randomly selecting background points for the point process models, background sampling was instead weighted by the distribution of IMR as a robust indicator of health system accessibility and infrastructure. Second, the species distribution models used to construct Ardeidae suitability were based on human observations and so are also subject to reporting bias, insofar as bird accessibility is likely to impact reporting effort. Reporting bias in Ardeidae observations was corrected by weighting the sampling of background points by HFP as an indicator of accessibility. In addition, although this study was able to estimate the landscape suitability of several Ardeidae species, there were some species for which there were too few observations to validly model suitability. As such, we concede that this work is not an exhaustive representation of all possible species niches and therefore some aspects may yet remain undescribed by these findings. Third, the climate measures interrogated in the models presented were based on decadal averages over the period from 1950 to 2000, which assumes homogeneity over this time period as well as over the period of JEV outbreak surveillance under investigation. However, the current study sought to model the influence of climate features in the landscape rather than specific weather events, so these assumptions were deemed appropriate.

This study showed that JEV risk in India was strongly associated with the distribution of animal hosts and the shared landscapes between fragmented rain-fed agriculture and river and freshwater marsh wetlands. Importantly, the convergence of livestock and wildlife hosts with ecotonal landscapes of rain-fed agriculture and wetland habitat may provide unique transdisciplinary opportunities to target distinct aspects of JEV landscape epidemiology across different sectors within local and state (or national) municipalities where limited and differential resource capacities will likely require collaborative effort for the optimal control of JEV outbreaks. This has logistical appeal as some interventions may be best suited to delivery at the local community level, whereas others may be more efficiently delivered by regional actors with greater resource availability. For example, the World Health Organization has outlined potential forms of landscape manipulation and modification, such as the rotation or synchronisation of crop cycles, alternating crop varieties with variable growing seasons or mechanical intervention on water movement through the landscape to subvert vector breeding.^46^ Moreover, the mitigation of vector abundance directly associated with rain-fed agriculture may be negated where wetland habitat is also present representing a refuge for mosquitoes from control measures and therefore a need for more direct monitoring of local ecotones and adjacent wetland ecosystem interiors. Alternatively, there may be opportunities for the repositioning of livestock animal pens at sites more distal to human residences, or locations of agricultural activity, to limit the vector–animal–human interface.^46^ These kinds of hyperlocal interventions could be ideally suited to administration by local municipalities such as the subdistrict taluks (tehsils), particularly since such interventions often require working closely with affected communities. In contrast, targeting landscape features that require more resource-intensive interventions such as broad livestock surveillance, or vaccination campaigns for humans (or livestock), may be more effectively orchestrated at the state or national levels. Furthermore, the coupling of habitat conservation with food security—a necessary and fundamental cooperative endeavour for the sustainable sharing of space between humans and wildlife in wetlands—will further require the collaboration of multiple sectors at local and state levels of organisational infrastructure. As a starting point, the current work highlights the importance of developing transdisciplinary, environmentally responsive JEV surveillance infrastructure for vectors, animals, humans and ecosystems to inform effective and equitable interventions that operate in genuine service to One Health.

## Ethics approval

Ethics approval was not required for this study because individual human participants were not included.

## Data availability

The data underlying this article are available at https://figshare.com/s/3f9777be3f569bc48ab7.

## Supplementary data

Supplementary data are available at *IJE* online.

## Author contributions

Conceptualisation: M.W., A.P., N.V., D.S., C.W., S.S., C.M.; methodology: M.W., A.P., N.V., S.S., C.M.; formal analysis: M.W.; investigation: M.W., A.P., N.V., D.S., C.W., S.S., C.M.; data curation: M.W.; writing—original draft preparation: M.W., A.P., N.V., D.S., C.W., S.S., C.M.; writing—review and editing: M.W., A.P., N.V., D.S., C.W., S.S., C.M.; supervision: C.M. All authors have read and agreed to the published version of the manuscript.

## Funding

None.

## Supplementary Material

dyac050_Supplementary_Data
