# Supplementary material for: High-risk landscapes of Japanese encephalitis virus outbreaks in India converge on wetlands, rain-fed agriculture, wild Ardeidae, and domestic pigs and chickens
Source: Int J Epidemiol. 2022 Mar 31;51(5):1408–18. doi: 10.1093/ije/dyac050 (PMC9557850; doi:10.1093/ije/dyac050)
Supplement: dyac050_Supplementary_Data [file dyac050_supplementary_data.zip › ije-2021-09-1340-File007.pdf]

Methods

Data sources

Human data

The National Centre for Disease Control's Integrated Disease Surveillance Programme (IDSP) maintains ongoing surveillance of JEV infections under the administration of India's Ministry of Health and Family Welfare[1]. There were 294 laboratory-confirmed and location-unique outbreaks of JEV reported at village level (spatial resolution of 1 arc minute, or approximately 2 km) to the IDSP between 1 January, 2010 and 31 December, 2020. These were included as the primary training data in the current study. As a test of the external validity of these surveillance data, a secondary dataset (n = 27) comprising all available independent, laboratory-confirmed community surveys of human and mosquito infection conducted within the same time period as the IDSP surveillance and with published location data were used to test the performance of models trained with the IDSP surveillance data[2–5]. Twenty-four of the 27 surveyed locations were based on population-based human sero-surveys (n = 12)[2], field-based entomological surveys (n = 3)[4], or a combination of entomological surveys and clinical-based human surveys (n = 9)[5], and were not in response to outbreaks. All of these locations were in rural settings and none of these locations corresponded to reported locations in the IDSP training data. One source[3], comprising a human sero-survey at four locations (one village could not be geo-located and therefore is not counted among the 27 locations in the testing dataset), was in response to an ongoing outbreak. Each of the three included locations also corresponded to rural landscapes. To test the influence of the locations from this latter study, model validation was compared with and without the three included in the testing data. The data underlying this article are available at Figshare (https://figshare.com/s/3f9777be3f569bc48ab7).

JEV infections often disproportionally affect communities of lower socioeconomic status with limited access to health care, so this study adjusted for potential reporting bias of JEV infections using

the distribution of health system performance as a representation of the local capacity to detect cases (see modelling description below). The infant mortality ratio (IMR) was chosen as a proxy for health system performance since it has been validated as representative of health infrastructure and health system performance and used to assess health service delivery and performance in diverse settings[6,7]. Moreover, the IMR is correlated with the Inequality-Adjusted Human Development Index (IHDI) and the Human Development Index (HDI) and is therefore an important representation of the economic, social, and environmental structural determinants of population health[6,8]. The raster of the IMR was obtained from the Socioeconomic Data and Applications Center (SEDAC) repository[9].

Human population density was derived from the Gridded Population of the World estimates for the 2010 population[10] to represent the baseline population at the beginning of the period under study. The raster data product was obtained from the SEDAC repository.

Animal data

The Global Biodiversity Information Facility (GBIF) was used to acquire all observations of Ardeidae species (241,784 individual observations of 15 species; Supplementary Tables and Figures: Supplementary Table S1; *Bubulcus coromandus* was previously considered a subspecies of *Bubulcus ibis*, but is now listed as a separate species by the International Ornithologists' Union; both binomials are recorded in the GBIF database and so the observations were not combined here to maintain continuity with the database) between 1 January 2010 and 31 December 2020 across India so each species' distribution could be modelled[11]. Pig density data were obtained from the Gridded Livestock of the World[12] (GLW). While pigs have been demonstrated as key amplifying hosts for JEV, some evidence suggests that poultry may also act as bridging hosts to human spillover in some settings[13–15], so we additionally included chicken density and duck density in these analyses, obtained from the same GLW source. For some regions of the world these data demonstrate non-negligible spatial heterogeneity in error. However in India, the estimates were adjusted by animal censuses at the 2nd and 3rd stage

administration levels, corresponding to the district and taluk, respectively, which represented a high level of data verification at a sub-state scale[12].

Due to potential differential accessibility, the background points used to model Ardeidae species distributions were weighted by the human footprint (HFP) (see modelling description below) to correct for potential spatial reporting bias in the observations of these birds. Human footprint raster data were acquired from the SEDAC registry[16] and quantified according to a 2-stage classification system[17]. First, a metric for human influence was constructed based on the following eight categories: (1) population density, (2) road proximity, (3) rail line proximity, (4) navigable river proximity, (5) coastline proximity, (6) artificial light at night, (7) rural versus urban location, and (8) land cover. These categories were scored and summed to generate the human influence index (HII), which ranges from 0 (absence of human impact) to 64 (maximum human impact). The ratio of the range of minimum and maximum HII in the local terrestrial biome to the range of minimum and maximum HII across all biomes, expressed as a percentage, is then calculated to produce the HFP metric[17].

Environmental data

The structure of water movement through the landscape was quantified using hydrological flow accumulation obtained from the Hydrological Data and Maps based on SHuttle Elevation Derivatives at multiple Scales (HydroSHEDS) information system (https://hydrosheds.cr.usgs.gov/), which is derived from elevation data of the Shuttle Radar Topography Mission[18]. Hydrological flow accumulation measures the quantity of upland area draining into each 500 × 500 m area.

Wetlands were classified using the surface water data from the Global Lakes and Wetlands Database[19]. Wetland types represented in the current study comprised: coastal wetland, river, lake, and freshwater marsh[20]. To quantify proximity to each wetland type, the proximity function in the QGIS geographic information system was used to create distance rasters for each wetland class[21]. The pixel values of these rasters represent the distance in kilometres between each wetland type and all

other pixels within the geographic extent under study. The distance rasters were then used to investigate the relationships between distinct wetland environments and JEV outbreaks.

Agriculture data were obtained from the Global Food Security Support Analysis Data (GFSAD) project to describe the geographic extent of crops that employ rainfed water distribution systems[22]. Two primary classes of rainfed agricultural systems were represented: dominant rainfed crops and fragmented rainfed crops. A third class, highly fragmented rainfed crops, was also available but was highly correlated and exhibited considerable overlap with fragmented rainfed crops and yielded very similar relationships, so this class was considered redundant and not included in this investigation. As with the wetland classes described above, distance rasters were created for both the dominant rainfed crop and fragmented rainfed crop classes using the proximity function in QGIS.

Climate data were obtained from the WorldClim Global Climate database[23]. This investigation examined seasonal measures of precipitation due to the distinct seasonal pattern in JEV outbreaks, as particularly marked by monsoon-associated precipitation. Accordingly, rasters for the mean driest quarter precipitation and wettest quarter precipitation, as well as the mean annual temperature, were used in this analysis. All raster data described above were obtained at 30 arc seconds resolution.

Statistical analyses

Ardeidae species distribution modelling: An ensemble approach comprising boosted regression trees (BRT), random forests (RF), and generalised additive models (GAM) was used to estimate the landscape suitability of each of the 15 Ardeidae species. Species distribution models (SDMs) based on the two machine learning frameworks (BRT and RF) partition the data space according to algorithms that optimise homogeneity among predictors and a response (e.g. species presence), whereby optimised decision trees are iteratively determined and can capture complex interactions between predictors[24–27]. Whereas the GAM framework fits multiple basis functions for smoothed covariates to allow for nonlinear relationships between outcomes and covariates [28,29]. Each SDM under the three distinct

modelling frameworks (BRT, RF, and GAM) was fit using five-fold cross-validation. To prevent artificial spatial clustering of observation data due to potential sampling bias, the data were thinned to include only one observation per pixel in the analysis (Supplementary Table S1). Mean annual precipitation, mean annual temperature, isothermality, and proximity to surface water comprised the environmental features included in the SDMs. These variables exhibited low correlation with each other (all Pearson's r < 0.5) and therefore their inclusion together in the models was justified. Model performance, based on the area under the receiver operating characteristic curve (AUC), and model fit, based on the deviance, were used to evaluate each of the three SDM frameworks (BRT, RF, and GAM) for each ardeid species. Subsequently, an ensemble landscape suitability was estimated for each species from the three SDM frameworks using their weighted mean, with weights based on AUC[30]. Potential spatial sampling bias in the GBIF database was further adjusted for by sampling background points proportional to the human footprint as a proxy for landscape accessibility. The landscape suitability for each species was modelled at a spatial resolution of 30 arc seconds (~1 km). Individual species are presented with their number of field observations (and thinned analytical observations) and model metrics in Supplementary Table S1.

After modelling the distributions of individual Ardeidae species' landscape suitability, a composite of ardeid suitability was calculated based on the mean of all individual species suitability distributions. The degree of niche overlap[31] between each individual species landscape suitability and the composite species suitability was evaluated to determine the extent of heterogeneity between the species-specific environmental niches. More specifically, niche overlap was assessed to determine (1) if heterogeneity was too extensive to justify a composite representation of Ardeidae species landscape suitability, (2) if a small number of ardeid species demonstrating divergent landscape suitability should be considered individually in concert with a composite representation of landscape suitability for the remaining species, or (3) if the species demonstrated sufficient overlap in their environmental niches to justify a composite representation of Ardeidae species landscape suitability alone. The sdm package[30]

in the R platform[32] was used for fitting each model and the derivation of the three-model ensembles to each species and the dismo package was used to compare niche overlap[33].

JEV outbreak modelling: The JEV outbreaks were fit as a point process using homogeneous and inhomogeneous Poisson models[34]. This framework allows for the assessment of spatial dependencies among the outbreaks and, where such dependencies are identified, these can be evaluated with respect to environmental features that may account for the observed dependencies.

First, JEV outbreaks were fitted as a homogeneous Poisson process, with conditional intensity,

$$\lambda(u,X) = \beta, \qquad [1]$$

where u designates the geographic locations of outbreaks, X, and β represents the intensity parameter. Intensity is defined as the number of points in a subregion of a defined geographic extent. The homogeneous Poisson model is the null model representing complete spatial randomness (CSR). Under CSR, the expected intensity is proportional to the area of the subregion under consideration[34], i.e., there is no spatial dependency.

Second, the model with the assumption of CSR was compared to an inhomogeneous Poisson process, which incorporates spatial dependency of the outcome (JEV outbreaks) into the model structure and has conditional intensity,

$$\lambda(u,X) = \beta(u). \qquad [2]$$

With this model, the intensity is represented as a function of the location, u, of the JEV outbreaks. The inhomogeneous Poisson model supported substantive spatial dependency in JEV outbreak intensity as this was a markedly better fit than the CSR model and also demonstrated significant divergence from CSR in the K-function (see results below). Given the identified spatial dependence in JEV outbreaks, simple and multiple inhomogeneous Poisson models with environmental features were fitted with conditional intensity,

$$\lambda(u,X) = \rho\,(Z(u)), \qquad [3]$$

where ρ is the parameter representing the association between the point intensity and the feature Z at location u. The models' background points were sampled proportional to IMR, as described above, to control for potential reporting bias in the JEV infection surveillance.

As above for the SDMs, the outbreak occurrences were thinned to prevent over-fitting of the models. The data were thinned so that no more than one event was included within each pixel (1 arc minute. In addition, the environmental covariates were aggregated to 1.0 arc minute using the aggregate function in the raster package with the mean function applied to climate, distance, and suitability rasters, and the sum function applied to the livestock count rasters. Human population density was included in all models as an offset so that the models appropriately represented epidemiological risk. The crude associations between JEV outbreaks and mean dry quarter precipitation, mean wet quarter precipitation, mean annual temperature, hydrological flow accumulation, proximity to each wetland type, proximity to rainfed agricultural systems, the composite landscape suitability of Ardeidae species, pig density, chicken density, and duck density were initially assessed individually with a separate simple inhomogeneous Poisson model (Supplementary Table S2). Features demonstrating bivariate associations with confidence intervals that did not include 0 were included as covariates in the multiple inhomogeneous Poisson models (Supplementary Figure S1, Supplementary Figure S2, Supplementary Figure S3). The features included as covariates in the multiple inhomogeneous Poisson models demonstrated low correlation (all values of the Pearson's r were ≤ 0.53) and so were deemed appropriate to be included together in the models. Since the focus of this study was to identify landscapes of JEV risk, the degree of proximity to wetland classes and rainfed agriculture, rather than the degree of remoteness from them, was similarly the focus of the interrogation of these particular landscape features. As such, only those wetland feature classes that demonstrated significant inverse associations (i.e. proximity) were included in the multiple point process models, whereas those demonstrating associations of remoteness from JEV outbreaks were not. In addition, the two wetland

classes associated with remoteness from JEV outbreaks (Supplementary Table S2) were also highly correlated with the classes that were associated with proximity to JEV outbreaks due to their opposite associations, so all of these wetland classes could not be included together in the models due to multicollinearity and were not considered in separate models since remoteness from outbreaks was not the focus. The associations between JEV outbreaks and landscape features were represented by relative risks, which were computed from the regression coefficients of the inhomogeneous Poisson models. Interaction between fragmented rainfed agriculture and the two dominant wetland types, river and freshwater marsh, were examined separately using a river-rainfed crops model and a freshwater marsh-rainfed crops model with a corresponding interaction term included in each model, respectively. In this way, the interaction between fragmented rainfed agriculture and both river and freshwater marsh wetlands was used to evaluate the impact of their shared landscapes on JEV risk. The Akaike information criterion (AIC) assessed model fit, while the AUC assessed model performance. Importantly, model performance was tested against an independent, laboratory-confirmed dataset derived from the community-based surveys described above. The use of independent data for testing model performance provides a test of the external validity of the results thereby improving model assessment considerably. Model selection was based on a comparison of the fit (based on AIC) of the full model to reduced model groups nested on three broad environmental domains (hydrogeography, animal hosts, and climate). These were also compared against a stepwise selection procedure with the full point process model to see if there was any divergence in model selection[35,36]. Assessment of K-functions fitted to the JEV outbreaks before and after point process modelling with the specified environmental features was used to determine if the selected features adequately accounted for the observed spatial dependencies. The R statistical software version 3.6.1 was used to perform the analyses[32]. Point process models were fitted and K-functions estimated using the spatstat package[35,36].

References

1. National Centre for Disease Control, Directorate General of Health Services, Ministry of Health and Family Welfare. In press. Integrated Disease Surveillance Programme(IDSP). See https://idsp.nic.in/ (accessed on 3 September 2020).

2. Balakrishnan A, Thekkekare R, Sapkal G, Tandale B. 2017 Seroprevalence of Japanese encephalitis virus & West Nile virus in Alappuzha district, Kerala. *Indian J. Med. Res.* **146**, 70. (doi:10.4103/ijmr.IJMR_1638_15)

3. Dwibedi B, Mohapatra N, Rathore S, Panda M, Pati S, Sabat J, Thakur B, Panda S, Kar S. 2015 An outbreak of Japanese encephalitis after two decades in Odisha, India. *Indian J. Med. Res.* **142**, 30. (doi:10.4103/0971-5916.176609)

4. Ramesh D, Muniaraj M, Samuel Pp, Thenmozhi V, Venkatesh A, Nagaraj J, Tyagi B. 2015 Seasonal abundance & role of predominant Japanese encephalitis vectors Culex tritaeniorhynchus & Cx. gelidus Theobald in Cuddalore district, Tamil Nadu. *Indian J. Med. Res.* **142**, 29. (doi:10.4103/0971-5916.176607)

5. Nyari N, Singh D, Kakkar K, Sharma S, Pandey SN, Dhole TN. 2015 Entomological and serological investigation of Japanese encephalitis in endemic area of eastern Uttar Pradesh, India. *J. Vector Borne Dis.* **52**, 321–328.

6. Reidpath DD, Allotey P. 2003 Infant mortality rate as an indicator of population health. *J. Epidemiol. Community Health* **57**, 344–346. (doi:10.1136/jech.57.5.344)

7. Choi J *et al.* 2019 Health Indicators Related to Disease, Death, and Reproduction. *J. Prev. Med. Public Heal.* **52**, 14–20. (doi:10.3961/jpmph.18.250)

8. Ignacio Ruiz J, Nuhu K, Tyler McDaniel J, Popoff F, Izcovich A, Martin Criniti J. 2015 Inequality as a

powerful predictor of infant and maternal mortality around the world. *PLoS One* **10**. (doi:10.1371/journal.pone.0140796)

9. Center for International Earth Science Information Network - CIESIN - Columbia University. 2019 Global Subnational Infant Mortality Rates, Version 2.

10. Center for International Earth Science Information Network - CIESIN. 2018 Gridded Population of the World, Version 4 (GPWv4).

11. GBIF. 2021 GBIF occurrence download - Ardeidae India. *Glob. Biodivers. Inf. Facil.* See https://doi.org/10.15468/dl.s99zmx.

12. Robinson TP *et al.* 2014 Mapping the global distribution of livestock. *PLoS One* **9**, e96084. (doi:10.1371/journal.pone.0096084)

13. Ogata M, Nagao Y, Jitsunari F, Kitamura N, Okazaki T. 1970 Infection of herons and domestic fowls with Japanese encephalitis virus with specific reference to maternal antibody of hen (epidemiological study on Japanese encephalitis 26). *Acta Med. Okayama* **24**, 175–184.

14. Bhattacharya S, Chakraborty S, Chakraborty S, Ghosh K, Palit A, Mukherjee K, Chakraborty M, Tandon N, Hati A. 1988 Density of Culex vishnui and appearance of JE antibody in sentinel chicks and wild birds in relation to Japanese encephalitis cases. *Trop. Geogr. Med.* **38**, 46–50.

15. Bhattacharya S, Basu P. 2014 Japanese Encephalitis Virus (JEV) infection in different vertebrates and its epidemiological significance: a Review. *Int. J. Fauna Biol. Stud.* **1**, 32–37.

16. Socioeconomic Data and Applications Center | SEDAC. In press. Methods » Last of the Wild, v2 | SEDAC. See http://sedac.ciesin.columbia.edu/data/collection/wildareas-v2/methods (accessed on 23 December 2014).

17. Sanderson EW, Jaiteh M, Levy MA, Redford KH, Wannebo A V, Woolmer G. 2002 The Human Footprint and the Last of the Wild. **52**.

18. Lehner B, Verdin K, Jarvis A. 2006 HydroSHEDS Technical Documentation.

19. Fund WW. In press. Global Lakes and Wetlands Database. See http://www.worldwildlife.org/pages/global-lakes-and-wetlands-database.

20. Lehner B, Döll P. 2004 Development and validation of a global database of lakes, reservoirs and wetlands. *J. Hydrol.* **296**, 1–22. (doi:10.1016/j.jhydrol.2004.03.028)

21. QGIS Development Team. 2009 QGIS Geographic Information System.

22. Thenkabail P, Teluguntla P, Xiong J, Oliphant A, Massey R. 2016 LP DAAC - GFSAD1KCM. *Glob. Food Secur. Support Anal. Data Crop Mask 2010 Glob. 1 km V001*.

23. WorldClim - Global Climate. In press. Data for current conditions (~1950-2000) | WorldClim - Global Climate Data. *WorldClim - Glob. Clim. Data*. See http://www.worldclim.org/current (accessed on 23 October 2014).

24. Elith J, Leathwick JR, Hastie T. 2008 A working guide to boosted regression trees. *J. Anim. Ecol.* **77**, 802–13. (doi:10.1111/j.1365-2656.2008.01390.x)

25. Friedman J. 2001 Greedy function approximation: a gradient boosting machine. *Ann. Stat.* **29**, 1189–1232.

26. Breiman L. 2001 Random forests. *Mach. Learn.* **45**, 5–32. (doi:10.1023/A:1010933404324)

27. James G, Witten D, Hastie T, Tibshirani R. 2000 *An introduction to Statistical Learning*. (doi:10.1007/978-1-4614-7138-7)

28. Wood SN. 2004 Stable and Efficient Multiple Smoothing Parameter Estimation for Generalized

Additive Models. *J. Am. Stat. Assoc.* **99**, 673–686. (doi:10.1198/016214504000000980)

29. Wood SN. 2017 *Generalized additive models : an introduction with R*. 2nd edn. New York: Chapman and Hall/CRC. See https://books.google.com.au/books?id=HL-PDwAAQBAJ&printsec=frontcover&dq=Wood,+S.N.+(2017)+Generalized+Additive+Models:+An+Introduction+with+R+(2nd+edition).+Chapman+and+Hall/CRC.&hl=en&sa=X&ved=0ahUKEwiB_43x9NvjAhUC4nMBHU7CDWQQ6AEIMDAB#v=onepage&q=Wood%2.

30. Naimi B, Araújo MB. 2016 sdm: a reproducible and extensible R platform for species distribution modelling. *Ecography (Cop.).* **39**, 368–375. (doi:10.1111/ecog.01881)

31. Warren DL, Glor RE, Turelli M. 2008 Environmental niche equivalency versus conservatism: quantitative approaches to niche evolution. *Evolution* **62**, 2868–83. (doi:10.1111/j.1558-5646.2008.00482.x)

32. R Core Team. 2016 R: A language and environment for statistical computing.

33. Hijmans RJ, Phillips S, Leathwick JR, Elith J. 2014 Package ‘dismo’. *Compr. R Arch. Netw.* , 1 – 65.

34. Baddeley A, Turner R. 2000 Practical Maximum Pseudolikelihood for Spatial Point Patterns (with Discussion). *Aust. <html_ent glyph=‘@amp;’ ascii=‘&’/> New Zeal. J. Stat.* **42**, 283–322. (doi:10.1111/1467-842X.00128)

35. Baddeley A, Turner R. 2005 spatstat: An R Package for Analyzing Spatial Point Patterns. *J. Stat. Softw. 12(6)*

36. Baddeley A, Rubak E, Turner R. 2015 *Spatial Point Patterns: Methodology and Applications with R*. CRC Press. See https://books.google.com/books?id=rGbmCgAAQBAJ&pgis=1.
