# Supplementary material for: High-risk landscapes of Japanese encephalitis virus outbreaks in India converge on wetlands, rain-fed agriculture, wild Ardeidae, and domestic pigs and chickens
Source: Int J Epidemiol. 2022 Mar 31;51(5):1408–18. doi: 10.1093/ije/dyac050 (PMC9557850; doi:10.1093/ije/dyac050)
Supplement: dyac050_Supplementary_Data [file dyac050_supplementary_data.zip › ije-2021-09-1340-File008.pdf]

Supplementary Table S1. Ardeidae species niche comparisons based on ensemble species distribution models. Each species listed represents that species' modelled suitability with the associated number of observations of the species in the field (and the number of species used for analysis after thinning in parentheses), model fit (deviance), model performance (area under the receiver operating characteristic curve (AUC)), and individual niche overlap with the composite landscape suitability.

**Ardeidae species**	**Number of field observations**	**Deviance**	**AUC (%)**	**Niche overlap (%)**
Ardea alba	18173 (11794)	0.83	89	99.7
Ardea cinerea	17774 (11006)	0.85	89	99.4
Ardea purpurea	16406 (9784)	0.78	91	99.6
Ardeola grayii	56018 (30755)	0.87	88	99.7
Bubulcus coromandus	290 (207)	0.87	88	98.3
Bubulcus ibis	53548 (32862)	0.91	87	99.6
Butorides striata	3398 (2576)	0.84	89	99.5
Dupetor flavicollis (*Ixobrychus flavicollis*)	1602 (1278)	0.77	91	98.7
Egretta garzetta	36332 (22326)	0.87	88	99.8
Egretta gularis	3081 (2050)	0.41	98	96.4
Egretta intermedia (*Ardea intermedia*)	22406 (14473)	0.82	89	99.8
Gorsachius melanolophus	112 (107)	0.54	94	88.6
Ixobrychus cinnamomeus	2151 (1810)	0.78	91	99.1
Ixobrychus sinensis	2300 (1739)	0.71	92	98.8
Nycticorax nycticorax	8193 (5473)	0.86	89	99.7

Supplementary Table S2. Crude, bivariate regression coefficients and 95% confidence intervals for the associations between Japanese encephalitis virus outbreaks and each landscape feature as derived from an inhomogeneous Poisson model with only the one feature included in the model. Note: Proximity to wetland and rainfed agriculture was the specific focus of these particular landscape features, so only those features of these two classes that demonstrated significant inverse associations were included in the multiple point process models.

**Landscape feature**	**AIC**	**Coefficient**	**95% confidence interval**	**p-value**
Null model	875.58			
**Climate**				
Mean dry quarter precipitation (10 mm)	531.30	0.14	0.10 – 0.17	<0.00001
Mean wet quarter precipitation (10 mm)	491.59	0.007	0.006 – 0.008	<0.00001
Mean annual temperature (Celsius)	554.43	-0.07	-0.09 – -0.05	<0.00001
**Hydrogeography and surface hydrology**				
Distance to freshwater marsh (km)	403.06	-0.005	-0.006 – -0.004	<0.00001
Distance to lakes (km)	573.37	0.005	-0.0006 – 0.010	0.05
Distance to rivers (km)	498.72	-0.008	-0.010 – -0.006	<0.00001
Distance to coastal marsh (km)	549.43	0.001	0.0009 – 0.002	<0.00001
Distance to any surface water (km)	573.43	-0.003	-0.006 – 0.001	0.08
Hydrological flow accumulation	575.25	0.00	-0.00003 – 0.00002	0.27
**Rainfed agricultural systems**				
Distance to major rainfed agriculture (km)	576.12	-0.001	-0.008 – 0.006	0.36
Distance to fragmented rainfed agriculture (km)	547.63	-0.014	-0.020 – -0.008	<0.00001
**Animal hosts**				
Ardeidae landscape suitability (%)	563.04	1.14	0.54 – 1.73	0.0001
Pig density (deciles)	334.77	0.42	0.35 – 0.48	<0.00001
Chicken density (deciles)	483.20	0.22	0.17 – 0.27	<0.00001
Duck density (deciles)	386.38	0.36	0.30 – 0.42	<0.00001

Supplementary Figure S1. Climate feature distributions.

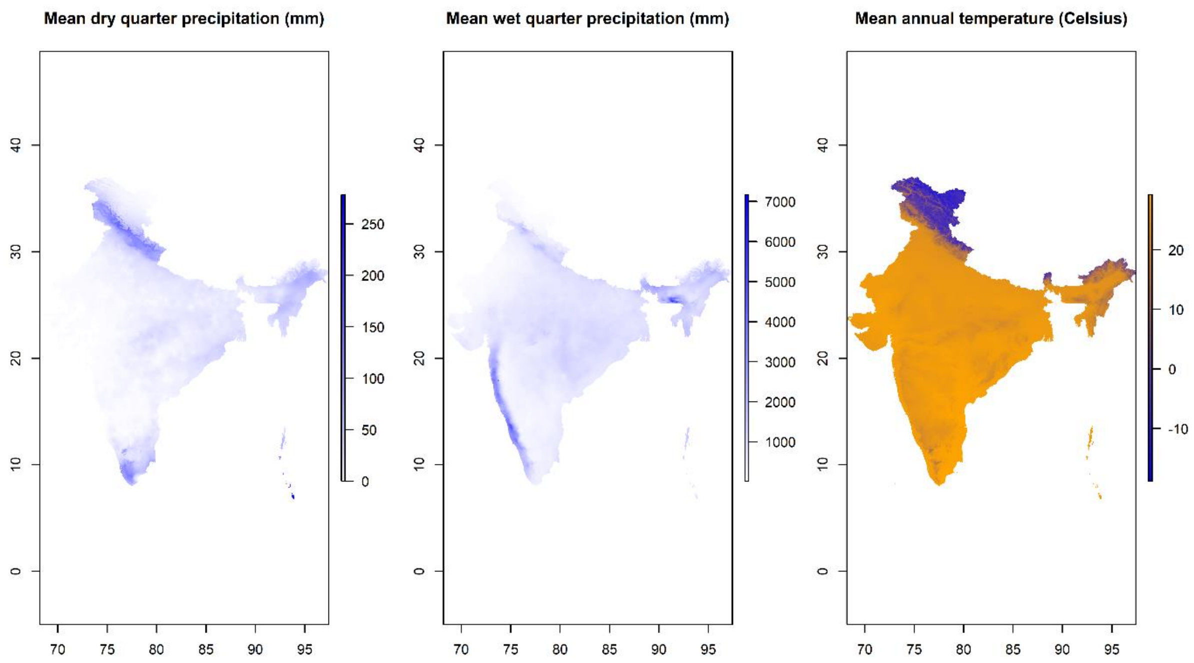

Supplementary Figure S2. Wetland and rainfed agriculture feature distributions.

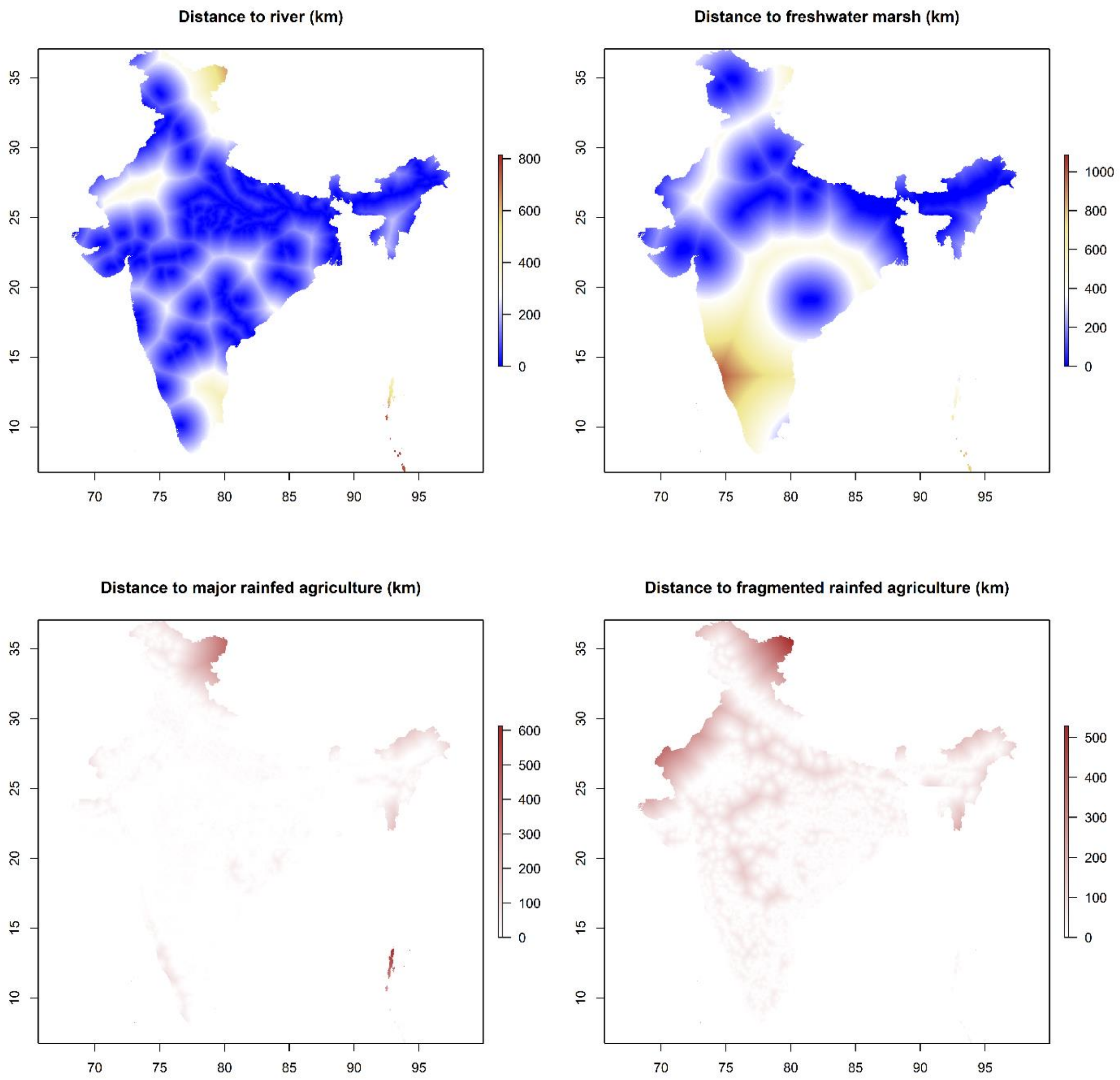

Supplementary Figure S3. Animal host feature distributions.

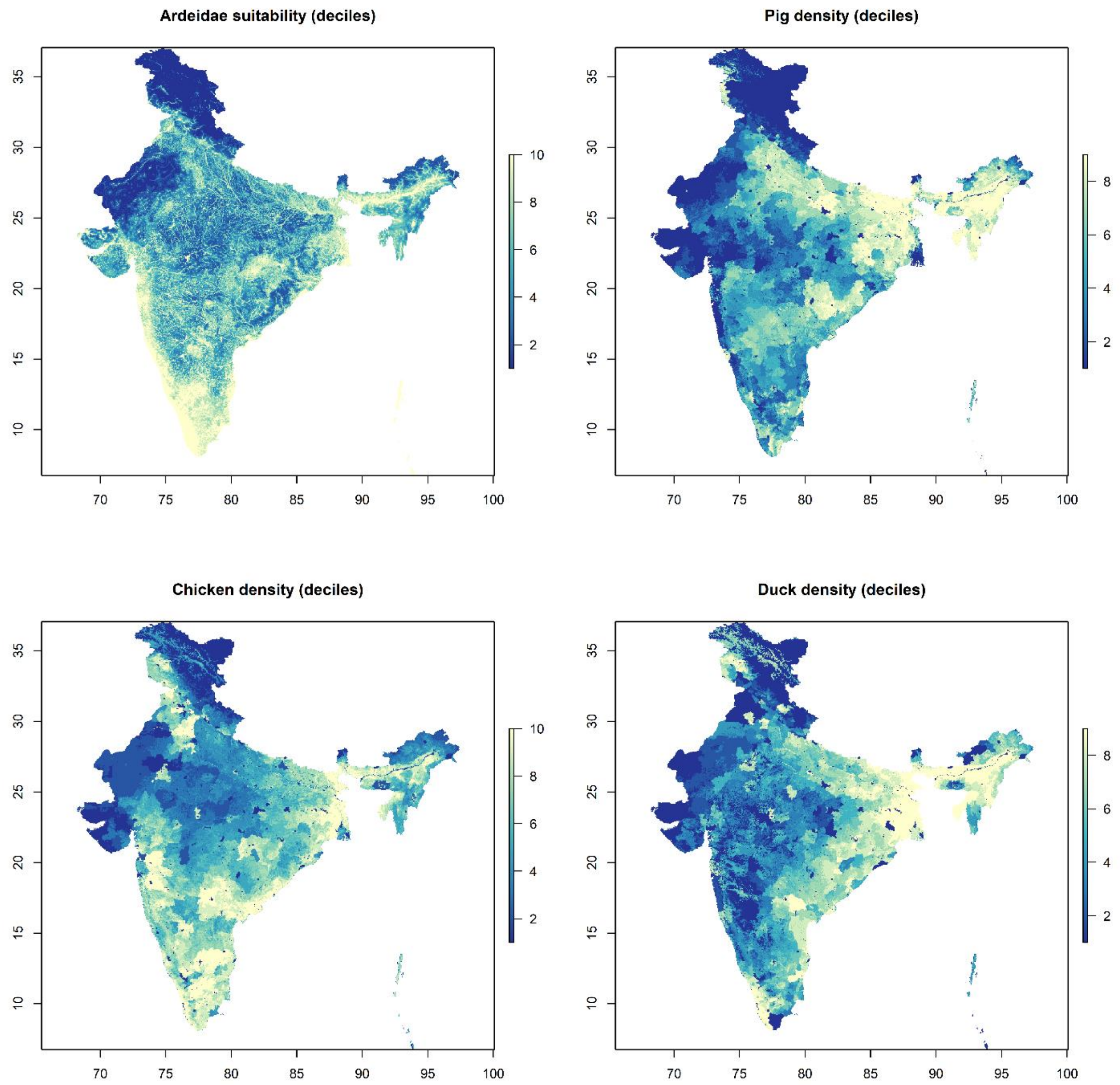

Supplementary Table S3. Japanese encephalitis virus (JEV) outbreak multiple inhomogeneous Poisson process model comparisons by Akaike information criterion (AIC) and area under the receiver operating characteristic curve (AUC). As a sensitivity analysis, model performance was re-evaluated for all models after excluding 3 survey locations from the independent testing data that were informed by an outbreak. The AUCs were thus recalculated and are represented in the table as AUC_{Sn}. Each nested multiple point process model includes those variables that were bivariately associated with JEV outbreaks (Supplementary Table S2).

Pont process models	AIC	AUC (%)	AUC_{Sn} (%)
**Model 1 (Climate only)**: *Mean wet quarter precipitation + mean dry quarter precipitation + mean annual temperature*	448.08	89.7	96.1
**Model 2 (Wetlands only)**: *Freshwater marsh proximity + river proximity*	400.56	79.3	79.8
**Model 3 (Reservoir hosts only)**: *Ardeidae suitability + pig density + chicken density + duck density*	281.57	92.7	94.1
**Model 4 (Full, no interaction)**: *Ardeidae suitability + pig density + chicken density + duck density + fragmented rainfed ag proximity + freshwater marsh proximity + river proximity + mean wet quarter precipitation + mean dry quarter precipitation + mean annual temperature*	95.64	94.3	95.1
**Model 5 (Full, #1)**: *Ardeidae suitability + pig density + chicken density + duck density + fragmented rainfed ag proximity + freshwater marsh proximity + rainfed ag:freshwater marsh interaction + river proximity + mean wet quarter precipitation + mean dry quarter precipitation + mean annual temperature*	84.83	93.5	94.1
**Model 6 (Full, #2)**: *Ardeidae suitability + pig density + chicken density + duck density + fragmented rainfed ag proximity + freshwater marsh proximity + river proximity + rainfed ag:river interaction + mean wet quarter precipitation + mean dry quarter precipitation + mean annual temperature*	91.51	93.7	94.4
**Model 7 (Final, #1)**: *Ardeidae suitability + pig density + chicken density + fragmented rainfed ag proximity + freshwater marsh proximity + rainfed ag: freshwater marsh interaction + river proximity + mean wet quarter precipitation + mean dry quarter precipitation*	82.82	93.7	94.3
**Model 8 (Final, #2)**: *Ardeidae suitability + pig density + chicken density + fragmented rainfed ag proximity + freshwater marsh proximity + river proximity + rainfed ag:river interaction + mean wet quarter precipitation + mean dry quarter precipitation*	89.21	93.8	94.4
